# Cyanobacteria—From the Oceans to the Potential Biotechnological and Biomedical Applications

**DOI:** 10.3390/md19050241

**Published:** 2021-04-24

**Authors:** Shaden A. M. Khalifa, Eslam S. Shedid, Essa M. Saied, Amir Reza Jassbi, Fatemeh H. Jamebozorgi, Mostafa E. Rateb, Ming Du, Mohamed M. Abdel-Daim, Guo-Yin Kai, Montaser A. M. Al-Hammady, Jianbo Xiao, Zhiming Guo, Hesham R. El-Seedi

**Affiliations:** 1 Department of Molecular Biosciences, Wenner-Gren Institute, Stockholm University, SE-106 91 Stockholm, Sweden; 2 Department of Chemistry, Faculty of Science, Menoufia University, Shebin El-Kom 32512, Egypt; eslamshaker566@yahoo.com; 3 Chemistry Department, Faculty of Science, Suez Canal University, Ismailia 41522, Egypt; saiedess@hu-berlin.de; 4 Institut für Chemie, Humboldt-Universität zu Berlin, Brook-Taylor-Straße 2, 12489 Berlin, Germany; 5 Medicinal and Natural Products Chemistry Research Center, Shiraz University of Medical Sciences, Shiraz 71348-53734, Iran; jassbiar@sums.ac.ir (A.R.J.); fh_esfand@yahoo.com (F.H.J.); 6 School of Computing, Engineering & Physical Sciences, University of the West of Scotland, High Street, Paisley PA1 2BE, UK; mostafa.rateb@uws.ac.uk; 7 School of Food Science and Technology, National Engineering Research Center of Seafood, Dalian Polytechnic University, Dalian 116034, China; duming@dlpu.edu.cn; 8 Pharmacology Department, Faculty of Veterinary Medicine, Suez Canal University, Ismailia 41522, Egypt; abdeldaim.m@vet.suez.edu.eg; 9 Laboratory of Medicinal Plant Biotechnology, College of Pharmacy, Zhejiang Chinese Medical University, Hangzhou 311402, China; kaiguoyin@zcmu.edu.cn; 10 National Institute of Oceanography & Fisheries, NIOF, Cairo 11516, Egypt; Coralreef_niof1@yahoo.com; 11 Institute of Food Safety and Nutrition, Jinan University, Guangzhou 510632, China; jianboxiao@jnu.edu.cn; 12 School of Food and Biological Engineering, Jiangsu University, Zhenjiang 212013, China; guozhiming@ujs.edu.cn; 13 International Research Center for Food Nutrition and Safety, Jiangsu University, Zhenjiang 212013, China; 14 Pharmacognosy Group, Department of Pharmaceutical Biosciences, Uppsala University, Biomedical Centre, P.O. Box 574, SE-751 23 Uppsala, Sweden

**Keywords:** cyanobacteria, clinical trials, antioxidant, antiviral, COVID-19, dietary supplements, biotechnological applications, total synthesis

## Abstract

Cyanobacteria are photosynthetic prokaryotic organisms which represent a significant source of novel, bioactive, secondary metabolites, and they are also considered an abundant source of bioactive compounds/drugs, such as dolastatin, cryptophycin 1, curacin toyocamycin, phytoalexin, cyanovirin-N and phycocyanin. Some of these compounds have displayed promising results in successful Phase I, II, III and IV clinical trials. Additionally, the cyanobacterial compounds applied to medical research have demonstrated an exciting future with great potential to be developed into new medicines. Most of these compounds have exhibited strong pharmacological activities, including neurotoxicity, cytotoxicity and antiviral activity against HCMV, HSV-1, HHV-6 and HIV-1, so these metabolites could be promising candidates for COVID-19 treatment. Therefore, the effective large-scale production of natural marine products through synthesis is important for resolving the existing issues associated with chemical isolation, including small yields, and may be necessary to better investigate their biological activities. Herein, we highlight the total synthesized and stereochemical determinations of the cyanobacterial bioactive compounds. Furthermore, this review primarily focuses on the biotechnological applications of cyanobacteria, including applications as cosmetics, food supplements, and the nanobiotechnological applications of cyanobacterial bioactive compounds in potential medicinal applications for various human diseases are discussed.

## 1. Introduction

Cyanobacteria, whose metabolism has played a unique role in ecosystems since ancient times, have probably been in existence for more the 3.5 billion years [1]. Cyanobacteria, previously known as blue-green algae, are the most primitive organism present on the earth. They play a vital role as the primary sources of oxygen and as nitrogen fixing agents in aquatic environments [2]. Indeed, the oxygen fixing properties of these organisms made life on Earth possible billions of years ago. Cyanobacteria are found in different habitats, from fresh water lakes, ponds to maritime coasts and the open ocean, occupying the largest ecosystem in the planet. The aquatic cyanobacteria are divided into two large ecological groups: planktonic cyanobacteria, which float freely in the water column, and benthic cyanobacteria, which adhere to submerged solid surfaces (i.e., sediments, rocks, stones, algae, and aquatic plants) [3]. For example, the planktonic cyanobacteria species *Prochlorococcus* and *Synechococcus* are prevalent in many oceans [4]; additionally, *C theyanobium* and *Synechocystis* genera are vastly distributed in marine planktonic collections [5]. Some researchers believe that the “Red Sea” has its name because of the dense population of *Trichodesmium erythraeum* (sea sawdust), which is mostly present there. In tropical seas with surface temperatures above 25 °C and saltiness up to 35%, *Trichodesmium* sp. occurs. *Trichosdesmium* is a filamentous nonheterocystous cyanobacterium, that fixes air N_2_ [6]. *Microcystis*, *Cylindrospermopsis*, *Anabaena* and *Aphanizomenon* are the common genera that flourish. Their environmental vulnerability and short life cycles leading to the rapid turnover of organisms promote their use as biological indicators for environmental studies [7]. For example, N_2_ cyanobacterial fixing was used to understand the quality of water with extremely high turbidity, the low N: P ratio, the toxicity of metals and the environmental limits of nitrogen. Some of these organisms and symbiotic systems like Azolla, particularly for rice production, are used as biofertilizer [8]. They are also used in oxidation ponds and in treating plants for waste and sludge [9]. Recently, a few species were investigated for the development of biofuel after they were found to be the most effective of all living organisms in converting solar energy. In addition, their simple genomic structure has allowed genetic engineering to produce biofuel strains [10]. Cyanobacteria also interact with limestone; one of the more intriguing aspects is the capacity of some strains (euendoliths) to penetrate directly into the carbonate substrate. Inhibition and gene expression analysis using the Mastigocoleus BC008 have shown that the uptake and transport of Ca^2+^ is guided by a sophisticated mechanism unrivaled between the bacteria, P-style Ca^2+^ ATPases [11]. There is much evidence to be found that endolithic stigma products such as Brachytrichia and Mastigocoleus derive nutrients from the surrounding rock or from the outside [12]. Symbioses occur between cyanobacteria and other marine organisms such as sponges, ascidians, lichens, dinoflagellates, euchiuroid worms and macroalgae. They act as nitrogen fixing agents and releasers of dissolved organic carbon that benefit their hosts, also producing defensive specialized metabolites that save their hosts from being attacked by predators. One of the major host organisms for cyanobacteria are sponges. The most abundant bacterial phylum found in the different sponges of the Persian Gulf were cyanobacteria, constituting more than 44% of their total phylum diversity [13]. This indicates an important ecological interaction between the cyanobacteria and sponges [14,15]. Lichens are symbiotic associations between fungi and photosynthetic algae or cyanobacteria. Microcystins are potent toxins associated with aquatic cyanobacterial blooms that are responsible for the poisoning of both humans and animals [16].

Around 450 compounds from marine cyanobacteria were identified, particularly from the genera *Lyngbya*, *Oscillatoria*, and *Symploca*. Around 58% of the cyanobacterial metabolites were derived from *Oscillatoria*, while 35% of these natural products belonged to *Lyngbya* [17]. They importantly produce a broad variety of bioactive compounds, including toxin metabolites with potential anticancer properties, and produce promising results for future research into the regulation of human carcinoma [18]. For example, apratoxin D, was isolated from *Lyngbya* sp., has strong cytotoxicity against human lung cancer cells [19]. Additionally, symplocamide A was isolated from the marine cyanobacterium *Symploca* sp. and has shown powerful cytotoxicity to neuroblastoma cells and lung cancer cells [20]. For instance, Kurisawa et al. [21] isolated three new linear peptides from *Dapis* sp. Furthermore, cyanobacteria have long been known to produce the most efficient chemical defense specialized metabolites from different classes of natural products such as lipopeptides, alkaloids, depsipeptides, macrolides/lactones, peptides, terpenes, polysaccharides, lipids, polyketides [22]. For doing so, they used plenty of enzymes, specialized for the biosynthesis of their basic skeletons and also tailoring enzymes for their modification [23]. The majority of cyanobacterial activity is essentially related to their lipopeptide content [24,25,26].

Marine cyanobacteria with various and adverse chemical products have attracted the attention of many scientists from different fields, in particular medicinal chemistry and pharmacology [27]. They possess significant biological properties including antibacterial, antifungal, anticancer, antituberculosis, immunosuppressive, anti-inflammatory, and antioxidant properties [28,29,30]. Cyanobacteria are rich in omega-3 fatty acids, such as eicosapentaenoic acid (EPA) and docosahexaenoic acid (DHA), which are known to prevent inflammatory cardiovascular diseases [31]. Many studies have shown that cyanobacteria produce compounds with increased pharmaceutical and biotechnological interest and have applications in human health with numerous biological activities and as a dietary supplement [32]. Polyhydroxyalkanoates (PHAs) are polyesters produced by many cyanobacterial strains, which can be used as a substitute for nonbiodegradable plastics. Most studies have shown that oil-polluted sites are rich in cyanobacterial consortia capable of degrading oil components by providing the associated oil-degrading bacteria with the necessary oxygen, organic matter and fixed nitrogen [33]. However, cyanobacterial hydrogen was regarded as a promising alternative energy source which is now available on the market [34]. In addition to these applications, cyanobacteria are also used as food, fertilizers [35], wastewater treatment, aquaculture, a source of pharmacologically important secondary metabolites [33]. Nanobiotechnological applications of marine cyanobacterial metabolites that have biomedical applications may provide a novel method to overcome the poor water solubility of hydrophobic marine natural drugs and use cyanobacteria for industrial and medicinal purposes [36]. Nanomedicine has made significant advances in the use of nanocarrier formulations to deliver therapeutic drugs and diagnostic agents to tumor/cancer sites [37]. The use of marine cyanobacteria in cosmetics, cosmeceutical formulations and thalassotherapy due to its bioactive components possesses many advantages, including the maintenance of skin structure and function, which have gained interest as a concern for modern societies. It is also linked to its ability to regenerate and protect itself against external environmental conditions [38,39]. Cyanobacteria could be incorporated into the health and wellness treatments used in thalassotherapy centers due to their high concentration of biologically active substances [40]. This review presents an overview focusing on the biotechnological applications, therapeutic properties and clinical uses of cyanobacteria and their metabolites in addition to introducing their synthetic bioactive compounds.

## 2. Preclinical and Clinical Trials of Metabolites from Marine Cyanobacteria

**Figure 1:**
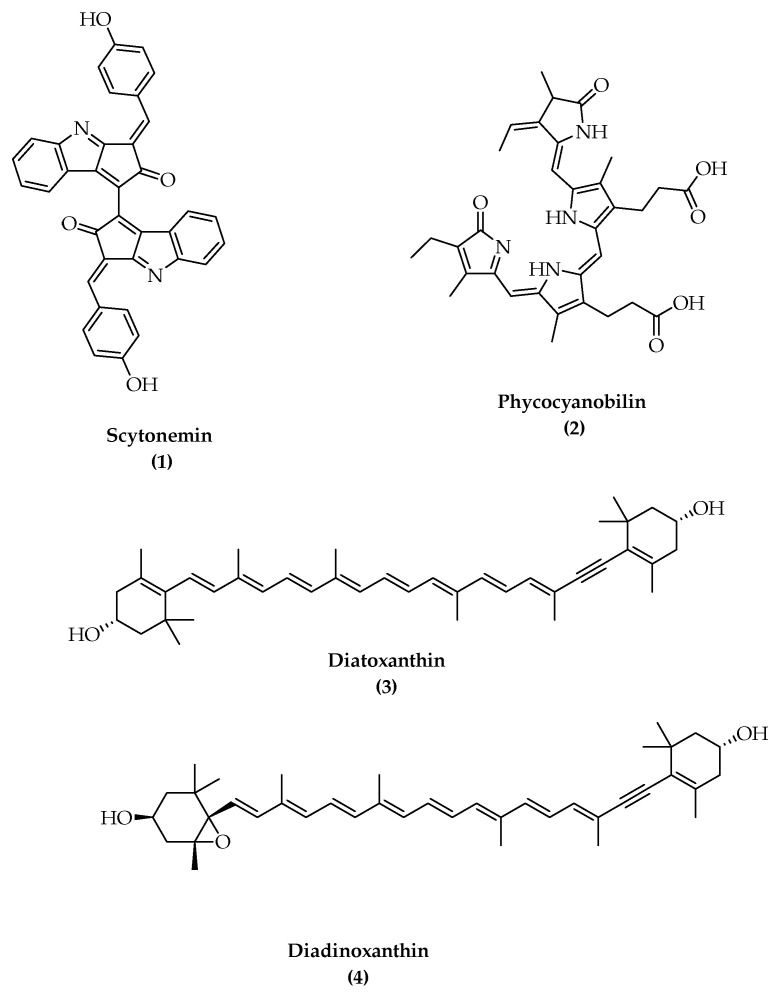
Antioxidant and antiobesity compounds from cyanobacteria.

**Figure 2:**
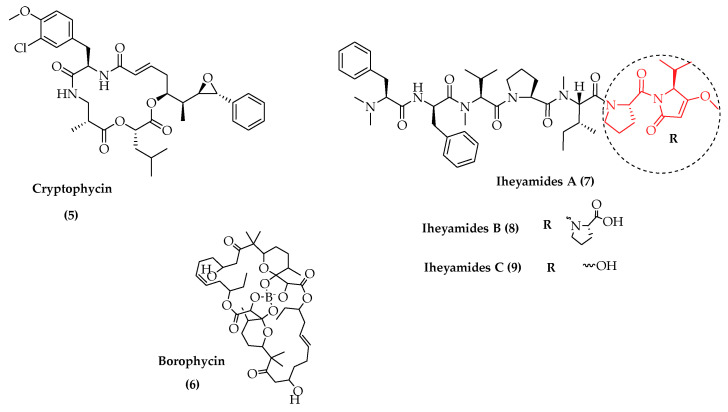
Cytotoxic compounds form *Nostoc* sp.

**Figure 3:**
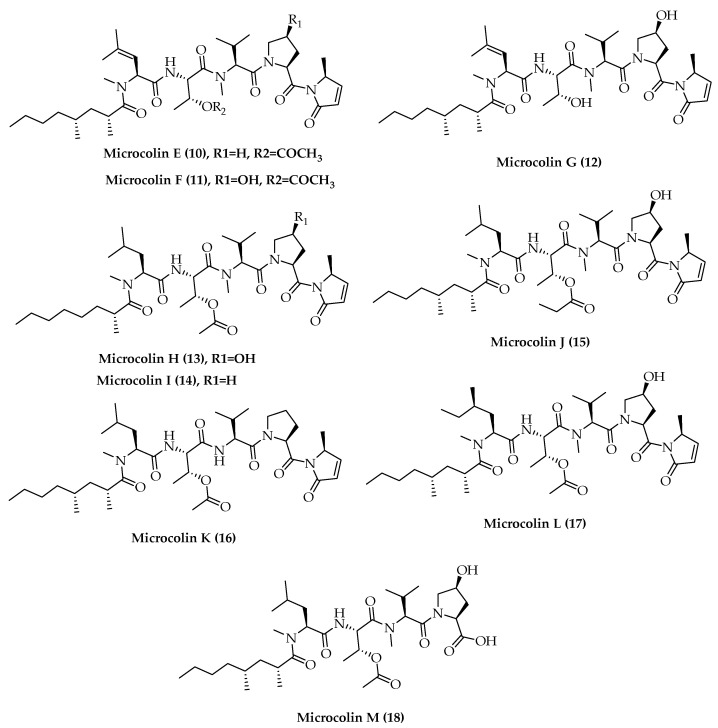
Cytotoxic compounds form *Moorea producens*.

**Figure 4:**
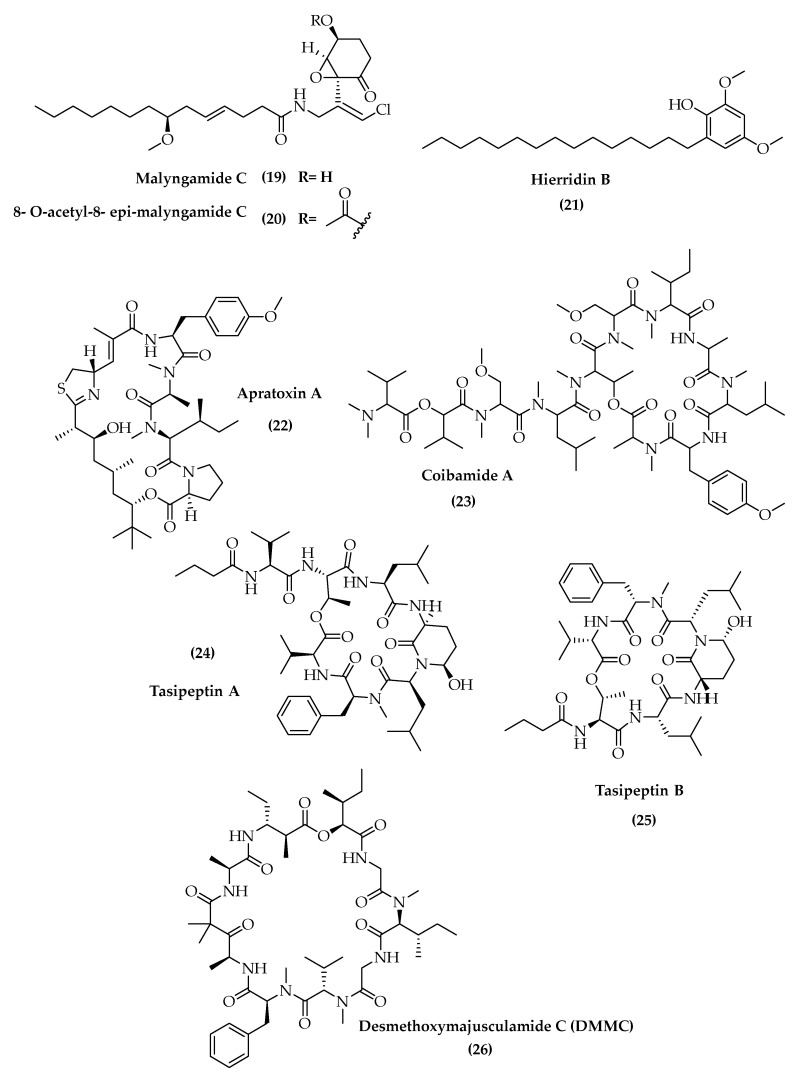
Cytotoxic cyanobacteria-derived metabolites.

**Figure 5:**
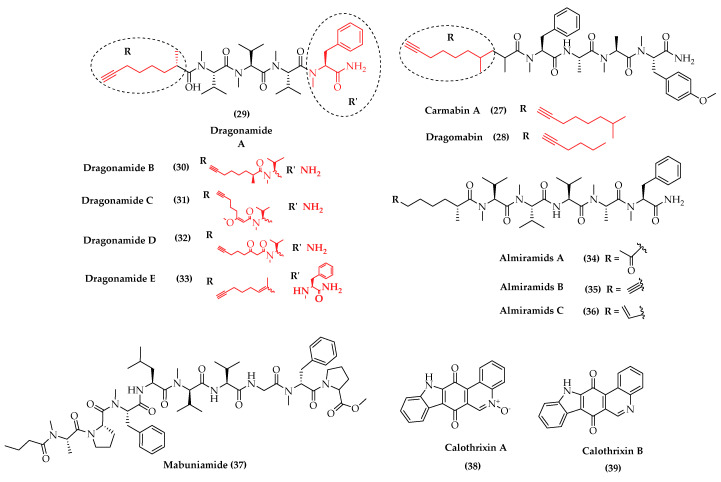
Antiparasite metabolites from cyanobacteria.

**Figure 6:**
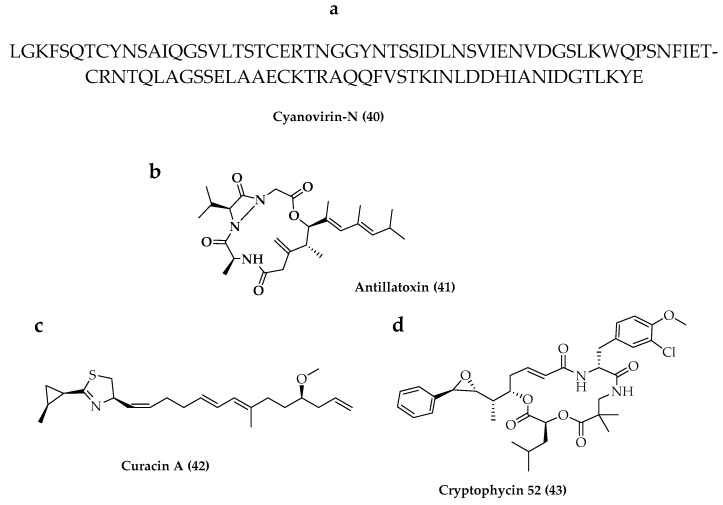
The structures of the cyanobacterial metabolites as drug leads against SARS-CoV-2, (**a**) the sequence of amino acids in **40**, (**b**) Antillatoxin (**41**), (**c**) Curacin A (**42**), (**d**) Cryptophycin **52** (**43**).

**Figure 7:**
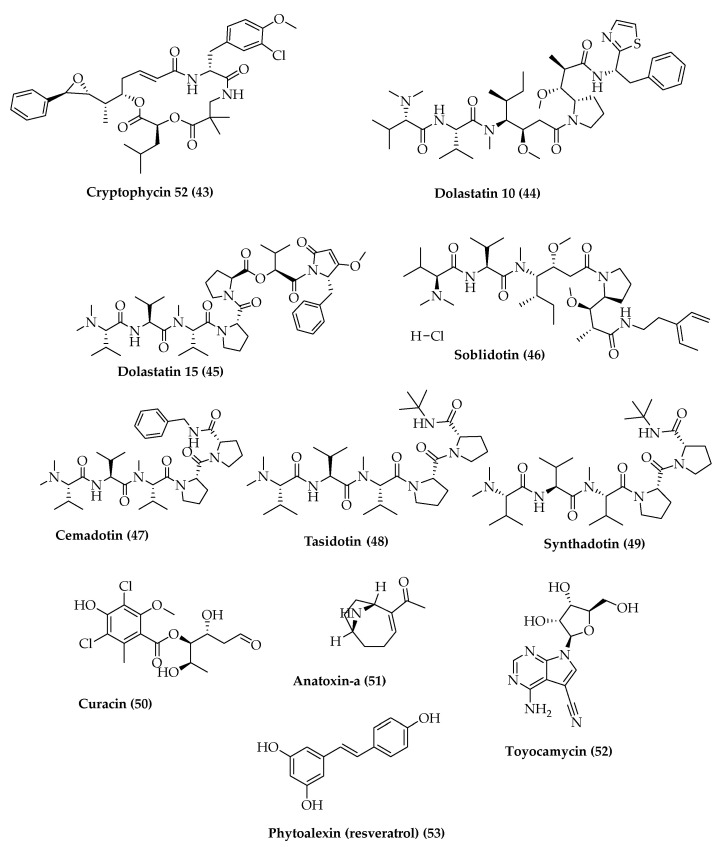
Clinically tested compounds and approved drugs from marine cyanobacteria.

The vital role of different metabolites from marine cyanobacteria as therapeutic agents is described and classified in two groups; preclinical and clinical entities. Those compounds (**1**–**41**, **43** and **44**) that are involved in preclinical trials are illustrated in Figure 1, Figure 2, Figure 3, Figure 4, Figure 5 and Figure 6. These bioactive compounds have well-known anti-inflammatory and anticancer properties and are used as external enzymes and antibiotics [41,42,43,44]. Those that are clinically validated, i.e., from compound **42**, **45**–**59**, are also mentioned in Figure 7. The different cyanobacteria species from which these metabolites have been reported and the related biological activities are discussed briefly.

### 2.1. Bioactive Constituents of Marine Cyanobacteria

#### 2.1.1. Antioxidant and Antiobesity Supplements from Cyanobacteria

Photosynthetic organisms such as cyanobacteria have developed many strategies to prevent the harmful effects of reactive oxygen species. Increased catalase and superoxide dismutase activity was necessary to regulate metal oxidative stress [45]. Scytonemin (SCY, **1**), a dimeric indole alkaloid which is therapeutic to the disorders of proliferation and inflammation, was isolated from *Lyngbya arboricola*, *Nostoc commune*, *Scytonema geitleri* [46]. *Rivularia* [47], and *Calothrix* sp. [48] showed strong antioxidant activity and averts up to 90% of solar UV radiation from entering the cell [49,50,51,52]. Cell safety can be provided by the enhancement of the antioxidant status and the elimination of superoxide anions and other oxygen derivatives [53,54]. In addition, antioxidant activity was reported from the methanolic extracts of *Synechocystis* sp., *Leptolyngbya* sp. and *Oscillatoria* sp. [55], and ethanolic extracts of *Nostoc* sp., *Anabaena* sp., *Calothrix* sp., *Oscillatoria* sp. and *Phormidium* sp. [56].

Phycocyanobilin (**2**) is tetraspyrole chromophore of blue green algae (*Spirulina*) which responsible for the blue color of *Spirulina*—in spite of that fact that it has almost the same structure as bilirubin, the pigment is more soluble than bilirubin, and **2** was reported to have proven health-promoting activities as an efficient quencher of different oxygen derivatives, and so possessed high antioxidant potential, protecting the live cell from extreme oxidative stress [57]. Spirulina is a cyanobacterium that can be used up orally, i.e., without any processing and is very useful to human health including enhancement of the immune system activity, antioxidant, anticancer, and antiviral effect. Thus, Spirulina is able to regulate hyperlipidemia and cholesterol levels and provide cell defense against a range of conditions including allergies, asthma, diabetes, hepatotoxicity, immunomodulation, inflammation and obesity [58,59].

Several clinical and preclinical trials have been conducted to test the benefits of *Spirulina* sp. on weight loss with promising results. Polyphenols are powerful antioxidants and natural products that may help reduce body weight. Miranda et al. [60] claimed that the main phenolic compounds—namely, chlorogenic acid, synaptic acid, salicylic acid, transcinnamic acid, and caffeic acid—were commonly present in Spirulina. The DPPH assay and hydroxyl scavenging assay done by Al-Dhabi and Valan Arasu [61], revealed that all the Spirulina extracts showed the activity in a concentration-dependent manner.

Yousefi et al. [62] studied 52 obese participants with a body mass index (BMI) > 25–40 kg/m^2^. They divided the candidates randomly into two different groups, namely, treated and placebo groups; the first group took Spirulina tablets (SP), 500 mg along with restricted calorie diet (RCD) 4 times a day, while the second group were given placebo tablets and RCD with the same daily regime for the 12 weeks of the intervention. Medical measurements, appetite scores and biochemical assessments were performed at the beginning, 6 and 12 weeks. Body weight, fat and BMI, together with waist dimension and appetite scores, were significantly reduced in the SP treated candidates compared to those measured in the placebo group.

Many pigments, such as carotenes, xanthophylls and chlorophylls, were identified using Fourier Transform Ion Cyclotron Resonance Mass Spectrometry (FT-ICR-MS), which was used to elucidate the qualitative profile of *Spirulina* (*Arthrospira platensis*). β-carotene, two xanthophylls (diatoxanthin (**3**) and diadinoxanthin (**4**)) showed the highest scavenging activity using precolumn reaction with DPPH radical followed by rapid UHPLC-PDA separation (Figure 1) [63]. 

#### 2.1.2. Cytotoxic Agents from Cyanobacteria

The peptolide cryptophycin (**5**), which was isolated from *Nostoc* sp., showed cytotoxic properties [64]. However, in other findings, the polyketide borophycin (**6**) from *Nostoc linckia* and *Nostoc spongiaeforme* showed antitumor activity against LoVo (MIC 0.066 µg/mL) and KB (MIC 3.3 µg/mL) [65,66]. Three new linear peptides (iheyamides A (**7**), B (**8**), and C (**9**)) were isolated from *Dapis* sp. (Figure 2). The new compounds were evaluated for cytotoxicity using normal human cells (WI-38), and antitrypanosomal activity against (*Trypanosoma brucei rhodesiense* and *Trypanosoma brucei brucei*) as models, respectively. The findings showed that compound **7** has potent antitrypanosomal and cytotoxic activity with IC_50_ values of 1.5 and 18 µM, respectively, compared with pentamide as a positive control with IC_50_ ranging between 0.001 to 0.005 µM. While compounds **8** and **9** had low activity with IC_50_ > 20 µM, the acting mechanism of **7** involved its growth-inhibitory activity against *T. b. rhodesiense* and *T. b. brucei*. Finally, the result indicates that compound **7** is a promising lead compound for a new drug [21].

Furthermore, Yu et al. [25] isolated nine new linear lipopeptides, microcolins E–M (**10**–**18**), from the marine cyanobacterium *Moorea producens*, which exhibited significant cytotoxic activity against lung carcinoma using MTT assay (Figure 3). Malyngamides are isolated amides of marine cyanobacteria. *Lyngbya majuscula*-producing malyngamide C (**19**) and 8-O-acetyl-8-epi-malyngamide C (**20**) have exhibited cytotoxicity against colon cancer cells HT29, with IC_50_ values 5.2 and 15.4 µM, respectively [67]. Additionally, they have antiproliferating effects against a variety of cancer cell lines, for example, heLa cell lines with EC_50_ (µM) 0.12 ± 0.01 and 0.24 ± 0.0, respectively [68]. Hierridin B (**21**) is a polyketide produced by *Cyanobium* sp. and has a selective cytotoxicity against colon cancer cell line HT-29 with an IC_50_ value of 0.1 µM [69]. Furthermore, apratoxins are cyclic depsipeptides isolated from marine cyanobacteria that inhibit several cancer cells lines at nanomolar concentration. Apratoxin A (**22**) produced by *Lyngbya boulloni* has been shown to be cytotoxic against adenocarcinoma cells [70]. Coibamide A (**23**) was isolated from *Leptolyngbya* sp. [71] and exhibited cytotoxicity against NCIH460 lung and mouse neuro-2a cells [72]. Tasipeptins A–B (**24**) and (**25**) are depsipeptides isolated from *Symploca* sp. that showed cytotoxic activity against KB cells with IC_50_ values of 0.93 and 0.82 µM, respectively [73]. Desmethoxymajusculamide C (**26**), DMMC is a cyclic depsipeptide from *Lyngbya majuscule* and showed potent cytotoxicity in both cyclic and ring-opened structural forms. Both of them showed cytotoxic activity against HCT-116 human colon carcinoma, H-460 human large cell lung carcinoma, MDA-MB-435 human carcinoma, neuro-2A murine neuroblastoma (Figure 4) [74].

#### 2.1.3. Antiparasite Agents

The bioactive linear alkynoic lipopeptides; carmabin A (**27**), dragomabin (**28**), dragonamide A (**29**) and dragonamide B (**30**) have been isolated from a Panamanian strain of the marine cyanobacterium *Lyngbya majuscula*. Good antimalarial activities of IC_50_ 4.3, 6.0, and 7.7 µM, were reported for the first three compounds, respectively, while the later **30** was inactive. Unlike its antimalarial effect, compound **30** exhibited the best cytoxicity against Vero cells (IC_50_ = 9.8 µM) among mammalian cells and parasites compared to that for **28** or **29** with IC_50_s = 182.3 µM and 67.8 µM, respectively [75]. Dragonamides C (**31**) and D (**32**), were isolated from *Lyngbya polychroa* [42], dragonamide E (**33**) from *L. majuscula* that was found to be active against leishmaniasis. Compound **29** and **33** exhibited strong antileishmanial activity with IC_50_ values of 6.5, 5.1, and 5.9 µM, respectively [76].

In 2010, Sanchez et al. [77] isolated and identified a series of cytotoxic lipopeptides from *L. majuscule*, namely almiramids A–C (**34**, **35**, and **36**), that revealed strong in vitro antiparasitic activity against genus *leishmania,* principally *L. donovani*, *L. infantum*, and *L. chagasi*. The lipopeptide mabuniamide (**37**) was isolated from *Okeania* sp. The evaluation of the antimalarial activity was conducted on *Plasmodium falciparum* 3D7 clone in in vitro. The results reported that **37** exhibits a potent effect with IC_50_ of 1.4 ± 0.2 µM when compared with positive control chloroquine (IC_50_ 7.6 ± 0.5 nM). This study records a flaw by not reporting the mode of action for the evaluated compound [26]. Calothrixins A (**38**) and B (**39**), as natural quinone products developed by *Calothrix* cyanobacteria, have also been shown to possess potent activity against malaria parasites; IC_50_ values were 58 ± 8 s.d. nM and 180 ± 44 s.d. nM, respectively, against *Plasmodium falciparum* [78] (Figure 5).

#### 2.1.4. Antiviral Natural Products with Anti-SARS-CoV-2 Potential from Cyanobacteria

Calcium spirulan (Ca-SP), a sulfated polysaccharide was isolated from *Arthrospira platensis*, is a promising candidate for the development of broad-spectrum antiviral drugs with novel modes of action. Ca-SP was found to be composed of rhamnose, 3-*O*-methylrhamnose (acofriose), 2,3-di-*O*-methylrhamnose, 3-*O*-methylxylose, sulfate, and uronic acids [79]. Ca-SP displays a broad-spectrum antiviral activity which was characterized by strong inhibition of in vitro replication of human viruses such as HCMV, HSV-1, HHV-6 and HIV-1 [80]. Polysaccharides possess significant antifibrotic properties in the pulmonary tissues and are considered beneficial against human coronavirus diseases. The polysaccharides derived from different species of *Spirulina* and especially *Spirulina platensis* were found to exhibit distinct antiviral activity against different enveloped viruses [81]. Hayashi et al. [80,81] evaluated the antiviral potential of calcium-spirulan derived from *Spirulina platensis* against HIV-1 and HSV-1 in comparison with the standard dextran sulfate. The serum samples of the mouse models administrated with calcium-spirulan showed long-lasting antiviral activity after 24 h of administration); however, their role in COVID-19 (SARS-CoV2 infections) remains limited [82]. The isolation of the antiviral polysaccharide nostoflan from a *Terrestrial Cyanobacterium* and *Nostoc flagelliforme* was another promising discovery, as it has potent antiherpes simplex virus type 1 (HSV-1) activity with a selectivity index (50% cytotoxic concentration/50% inhibitory concentration against viral replication) [83]. Using molecular docking and MD simulation studies, cyanovirin-N (**40**) was the highest among other lectins and was characterized with glycan type of S glycoprotein of SARS-CoV-2. Lokhande et al. [84] showed that BanLec wild-type and its mutant form have more thermodynamically stable binding complexes with SARS-CoV-2 S glycoprotein. By using in silico molecular docking and in vitro enzymatic assay screenings, it was found that **2** is a potent phytochemical inhibitor to SARS-CoV-2 M^pro^ and PL^pro^ proteases. Compound **2** demonstrated IC_50_ values of 71 and 62 μM for SARS-CoV-2 M^pro^ and PL^pro^, respectively. Further docking studies on compound **2** with other CoVsM^pro^ and PL^pro^ proteases revealed its broad-spectrum inhibition activity [85]. Naidoo et al. [86] examined 23 cyanobacterial metabolites against the SARS-CoV-2 M^pro^ and PL^pro^ proteases that were proved effective, i.e., antillatoxin (**41**), **38**, curacin A (**42**), **5**, cryptophycin 52 (**43**) and **22**. Compounds **22** and **43** showed superior inhibitory potential against SARS-CoV-2 M^pro^ based on the binding energy scores of the interactions. Compounds **5** and **43** displayed significant inhibitory prospects against the PL^pro^ of SARS-CoV-2 (Figure 6).

### 2.2. Clinical Trials of Metabolites from Marine Cyanobacteria

Focusing on marine biotechnology, more than 300 nitrogen-containing secondary compounds have been reported from the prokaryotic marine cyanobacteria [22]. Most of these metabolites are biologically active and are either nonribosomal (NRP) or derived from mixed polyketide-NRP biosynthetic pathways. NRP biomolecules and structural types of hybrid polyketides-NRPs are important components of natural products used as therapeutic agents. These include vancomycin, cyclosporine and bleomycin as antibiotics, immunosuppressive and anticancer agents [87]. Crude cyanobacterial extract screening has reported the effectiveness of identifying profitable compounds and been applied to clinical trials phases [88]. For example, the methanolic extracts of *Oscillatoria acuminata*, *Oscillatoria amphigranulata* and *Spirulina platensis* showed strong activity such as cytotoxicity, antioxidant and antimicrobial activity [89]. A remarkable drug discovery effort is made by the diversity of unique classes of marine cyanobacteria natural products [90]. Some of the marine cyanobacterial compounds and their analogs have shown exciting results and were successfully used in the clinical trials as shown in Table 1 (Preclinical, Phase I, Phase II, Phase III and IV), such as dolastatin 10 (**44**), dolastatin 15 (**45**), **43**, soblidotin (**46**), cemadotin (**47**), tasidotin (**48**), synthadotin (**49**), curacin (**50**) [91], anatoxin-a (**51**), bacteriocins, toyocamycin (**52**), phytoalexin (**53**), **40** and phycocyanin (**54**), and as various potential drug candidates for drug discovery. Their structures were exhibited in Figure 7, while their occurrences and bioactivities were reported in Table 1. 

Compounds **44** and **45** have exhibited promising results in phase II clinical trials for cancer treatments, while compounds **47**, **48** and **49**, as synthetic analogs of compound **45**, showed promising results in phase II clinical trials, as described in Table 1. Additionally, the synthetic analog **43**, which was applied to Phase III clinical trials to treat hypertension metabolic disorder, was derived from **5**. Compound **46**, a synthetic analog of compound, exhibited promising results in phase II clinical trials for sarcoma, melanoma and lung cancer treatment [92,101]. Compound **51** is a toxin isolated from blooms of the cyanobacterium *Anabaena circinalis* that is known for its worldwide production of a range of toxins [122]. The water samples were collected from east to west side of Zemborzycki reservoir, the samples were fixed with NaN_3_ and extracted by ultrasonication on ice in 75% methanol acidified with 2 M HCl. The tested scum extract was highly toxic when tested against the ciliate *Tetrahymena thermophile.* A complete growth inhibition was observed after 24 h of incubation with undiluted extract and the diluted ones that contain ≥ 258.90 µg/L of anatoxin-a [123].

Compound **40** is a 11-kDa virucidal protein isolated from the cultures of *Nostoc ellipsosporum* [124]. Filtration, freeze drying and extraction by MeOH-CH_2_Cl_2_ (1:1) followed by H_2_O were carried out to harvest the unialgal strain of the *N. ellipsosporum* cellular mass [125]. Buffa et al. [126] reported that compound **40** as a potent HIV type 1 inhibitor. The virus causes infection in cervical explant models with an IC_90_ of 1 mM. Dendritic cells were seen migrated out of the tissue explant and the secondary virus dissemination was inhibited by 70% when using the above described concentration.

Compound **52** and its derivatives are majorly responsible for the cytotoxicity and antifungal activity of the blue-green algae belonging to the scytonemataceae. Compound **52** was first isolated from *streptomyces tubercidicus* and *streptomyces toyocaensis*, respectively [127]. Compound **52** also was prescribed to induce a growth inhibition in pancreatic cancer cell lines by inhibiting the unfolded protein response, and also by the inhibition of both P-TEFb and PKC [128,129].

Bacteriocin is a genome mining study which proved the widespread of gene clusters encoding bacteriocins in cyanobacteria viz., *Prochlorcoccus marinus*, *Synechococcus* sp., *Cyanothece* sp., *Microcystis aeruginosa*, *Synechocystis* sp., *Arthospira* sp., *Nostoc* sp., *Anabaena* sp., *Nodularia* sp. [116,130]. Bacteriocins are defined as ribosomally synthesized proteinaceous compounds that are lethal to bacteria [131], in vivo activity following an intravenous regimen against pathogens, i.e., nisin has been shown to be 8–16 times more active than vancomycin in targeting *Streptococcus pneumoniae*. Equally important, nisin F, the naturally known nisin variant, was proved effective in stopping the pathogen growth in the respiratory system and the peritoneal cavity of the rat model, similarly suppressing the growth of *Staphylococcus aureus* in vivo when applied within bone cement [132].

Compound **54** extraction was evaluated using different solvents, including 10 mM sodium acetate buffer (pH 5.0), NaCl 0.15 M, 10 mM sodium phosphate buffer (pH 7.0), distilled water, and CaCl_2_ 10 g L^−1^. We mixed 2 g of dried biomass with 50 mL of the solvent and it then was subjected to shaking at 30 °C and extraction.

*Spirulina* is a blue-green alga that was used by NASA as a dietary supplement in space for astronauts. It has been reported that *Spirulina* exhibits anti-inflammatory properties by inhibiting the release of histamine from mast cells [133]. Ishii et al. [133] also studied the influence of *Spirulina* on IgA levels in human saliva and suggested a pivotal role of microalga in mucosal immunity.

Phytoalexin, resveratrol (53 is a stilbene compound; transresveratrol is synthesized in *Synechocystis* sp. PCC 6803 [134]. Resveratrol intake enhanced the release of the insulin-dependent glucose transporter, GLUT4, in rats with streptozotocin-induced diabetes and stimulated the insulin sensitivity mediated by the increase of adiponectin levels.

Furthermore, resveratrol induces the secretion of the gut incretin hormone glucagonlike peptide-1, as well as activating Sir2 (silent information regulatory 2) [135]. Furthermore, a phase II study of glembatumumab vedotin (GV) showed peptide for its active efficacy in the treatment of breast cancer and melanoma at a maximum tolerable dose of 1.0–1.88 mg/kg [136]. Brentuximab vedotin 63 (Adcetris™) (Adcetris as a trade name), peptide drug isolated from *Symploca hydnoides* and *Lyngbya majuscule* was approved by U.S. Food and Drug Administration (FDA) and European Medicines Evaluation Agency (EMEA) for cancer treatment [137].

## 3. Applications of Cyanobacteria in Biotechnology

**Figure 8:**
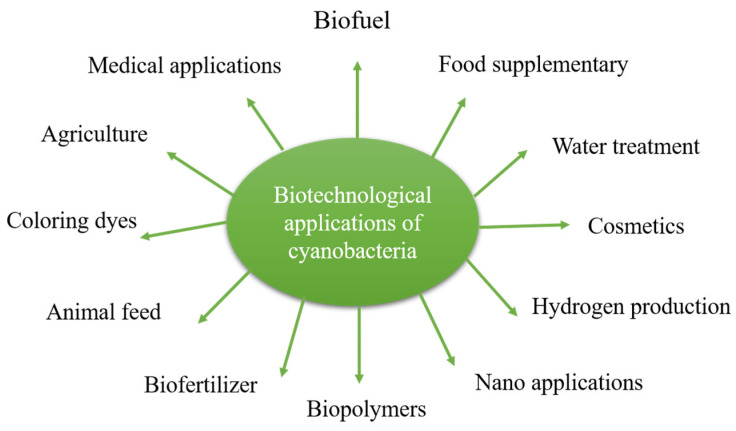
Biotechnological applications of cyanobacteria.

Cyanobacteria are arguably the most important group of microorganisms on the Earth. They are one of the early settlers of the barren parts of many oceanic regions [138]. Cyanobacteria fulfill vital ecological functions in the world’s oceans, being important contributors to global carbon and nitrogen budgets [139]. Recently significant attention has been paid to the application of marine cyanobacteria in the biotechnology field [92,140]. Due to their large range of industrial applications, they have been the focal point of many recent studies: biofuels, coloring dyes, food additives, and biofertilizers [141]. In addition, they are used in production of bioplastics, water treatment [33], hydrogen production [142], cosmetics [40], forestry, animal feed [143], and application in nanobiotechnology [36], as illustrated in Figure 8. Bioethanol, biodiesel, biohydrogen, and biogas are the highly in demand as energy sources [144]. Furthermore, the comparative yields of the biofuels produced by cyanobacteria and microalgae and other natural sources were reported [145]. The productivity of cyanobacteria and microalgae was 60,548 compared to palm seed, castor, sunflower, rape seed, soybean and the corn (the least productive source) with productivity of 4747, 1156, 946, 862, 321 and 152 (kg/ha year), respectively. The abovementioned information suggested the importance of the applications of cyanobacteria in biotechnology.

### 3.1. NanoBiotechnological Use of Cyanobacterial Extracts and Metabolites

**Figure 9:**
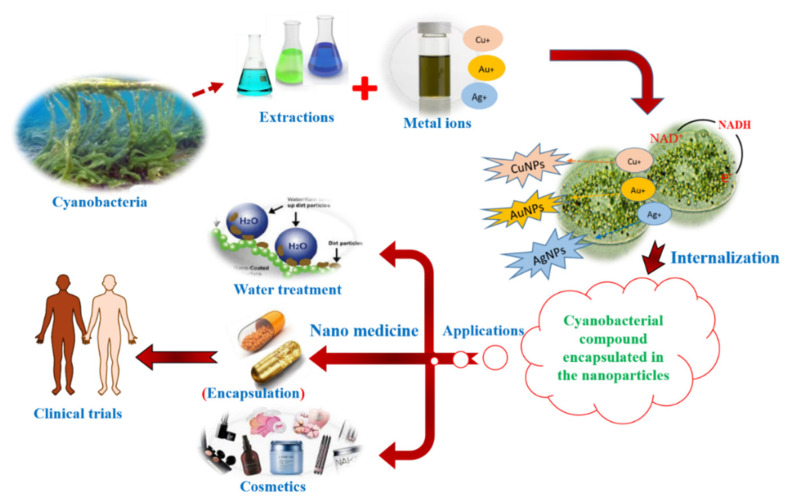
Applications of marine cyanobacteria in nanobiotechnology.

Nanoscience and nanotechnology have now become a modern discipline with a wide variety of applications for fundamental science. Nanotechnology plays a major role in multilayer trends, particularly in the health and life sciences, with a focus on ecofriendly new techniques [146]. Nanotechnology can encourage a new way to prevent hydrophobial, naturally occurring marine medicines with low water solubility [147] using various microorganisms, including simple bacteria and highly complex eukaryotes [148]. Nanotechnology is one of the fastest medical and industrial platforms [36] which could be implemented using desirable methods which improve stability, bioavailability and solubility [149]. Metallurgies, polysaccharides, lipids, peptide-based nanoformulations that play important role in medical diagnosis, drug delivery systems, antisense and gene therapies and tissue engineering, are healthy and ecofriendly nanomaterials [150,151]. In the fields of antimicrobial activity, wound care, medication transmission, the transmission of genes, cancer therapy and tissue engineering, polysaccharide-dependent nanoparticles are important components [152,153]. Marine cyanobacteria have many applications in nanobiotechnology, either through their direct use in the production of nanoparticles of different metals or through the nanotechnological processing of their bioactive metabolites in medicine as shown in Figure 9.

Several cyanobacterial species such as *Anabaena* sp., *Lyngbya* sp., *Synechococcus* sp., *Synechocystis* sp., *Cylindrospermopsis* sp., *Oscillatoria willei* and *Pectonema boryanum* were incorporated in the production of silver nanoparticles (NPs) by adding AgNO_3_ into a cell-free culture liquid prior to the cyanobacteria live and washed biomass suspension [154,155,156]. Cyanobacteria of the genera *Anabaena*, *Calothrix* and *Leptolyngbya* are also used to modify the shape of nanoparticles of gold, silver, palladium and platinum [157]. Such metal nanoparticles possess antimicrobial effects against many bacteria, including *Bacillus megatarium, Escherichia coli*, *Bacillus subtilis*, *Staphylococcus aureus*, *Pseudomonas aeruginosa*, and *Micrococcus luteus* [155]. In many areas, silver nanoparticles are also important, particularly in cosmetics and impregnating medical devices such as surgical masks and implantable devices with considerable antimicrobial effectiveness [158]. Encapsulation is by several coatings including external silicon layers of the cyanobacterial strain (*Synechococcus* sp.) [159].

Nanoformulated antiaging, antioxidants and anti-inflammatory creams or medicines have been developed with cyanobacterial secondary metabolites [32,40]. Nanoformulations of anticancer agents were also provided owing to simplifying delivery in a diversity of cancer states [155,160].

**Figure 10:**
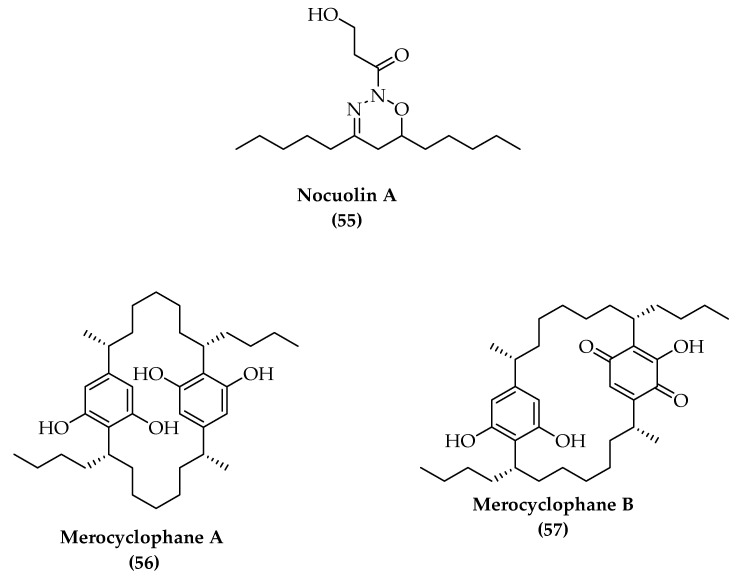
Marine cyanobacterial derived compounds suggested to be tested as nanoparticles.

There are a lot of bioactive substances that have been isolated from different cyanobacteria species such as *Lyngbya arboricola*, *Nostoc commune*, *Scytonema geitleri* [46]. *Rivularia* [47], and *Calothrix* sp. [48] such as compounds **1** and nocuolin A (**55**), merocyclophane A (**56**) and B (**57**) also have anti-inflammatory and anticancer activities, but there is no detailed work on their nanoparticle applications in drug developments. This may be done in future research works with the application of biotechnological or synthetic approaches to the mass production of such compounds to be tested as nanoparticles in in vivo tests (Figure 10).

### 3.2. Cyanobacteria: Foes or Friend of Skins, Their Use in Cosmetics

Cyanobacteria produce toxic metabolites and are allergens that have negative effects on the health of human skin. Despite the fact that cytotoxicity and poisoning were seen with some cyanobacterial genera, this adverse effect was potentially described for anticancer applications, for instance some toxins that combat the progress of human adenocarcinomas [18]. Furthermore, recent studies showed that some compounds, like the carotenoids phytoene, phytofluene and astaxanthin, can play healing roles and have antiaging effects for skin’s health and appearance and are used in cosmetics [161]. Non Melanoma Skin Cancer (NMSCs) have increased since the past two decades. Sunscreen is recommended in these cases by healthcare specialists [162]. The exploitation of cyanobacteria’s applications in sunscreens and cosmetics is warranted owing to their abilities to protect skin and prevent UV radiation damage. New formulations of large scale production in the cosmetics industry contain mycosporine and mycosporinelike amino acids (MAAs) and their derivatives due to their maximum absorption in UV range [163,164,165]. Skin bleaching, as a parameter of beauty, has become common all over the world, mainly in Asia [166]. Tyrosine kinase inhibitors perform the best for this purpose. This enzyme catalyzes the rate-limiting step of pigmentation. We summarized the cyanobacterial bioactive compounds which are so far used in cosmetics and skin protection.

The synthetic chemicals in cosmetics can be very harmful and may be toxic to the skin and cause aging, in addition to their high costs. Consumer tastes also affect the cosmetics industry. Compared to traditional cosmetics, the natural cosmetics industry remains a smaller fraction of the market [167]. Compared to synthetic cosmetics, herbal beauty products are mild, biodegradable, safe and have few low side effects [168]. Beside cosmetics, there is another terminology called “cosmeceuticals”, which are cosmetic products with active ingredients that exert a pharmaceutical therapeutic benefit [39]. Cyanobacteria contain a wide variety of bioactive health defense molecules [40], including flavonoids, pigments (e.g., β-carotene, c-phycoerythrin, phycobiliproteins), phenols, saponins, steroids, tannins, terpenes and vitamins [39]. These active metabolites lead researchers to check their skin care function.

The testing of cosmetic products will continue to be carried out in compliance with the current adopted guidelines and keys to health and effectiveness testing that can be reproducibly and scientifically verified [169]. Herbal cosmetics can be used for a long time to improve skin’s appearance and enhance skin gloss [170]. There are several causes which decrease the brightness of the skin, such as damage to DNA [171] caused by free radicals [172] which damages the skin and increases the risk of aging [173]. The antioxidants of free radical scavenging and reactive oxygen species must also be tested [174]. Other causes may be ageing, including chronic inflammation [175], which reduces skin brightness and may also contribute to skin cancer [176]. Therefore, the molecules must be investigated as antiaging and tested for anti-inflammatory activity. The molecules should be tested as sunscreen protective devices for sun blocking, causing DNA damage, skin aging and tumorigenesis [177]. Desiccation is extremely hazardous to skin and thus hydrating agents are very useful for skin care and treatment [178].

In cosmetic applications, there are only a few reports of cyanobacteria; some molecules from different species of cyanobacteria show positive results in skin-care such as methanolic extracts of exopolysaccharides from *Arthrospira platensis* used as antioxidant [40]. SCY (**1**), an indol alkaloid pigment, synthesized by many strains of cyanobacteria, also used as a defender sunscreen [47,51], isolated from the terrestrial cyanobacterium communal of *Nostoc* and supported its free radical scavenging activities [50]. Numerous clinical and preclinical trials found that *Spirulina* possesses antioxidants, immunomodulators and anti-inflammatory activities which protect against oxidative stress by preventing and inhibiting lipid peroxidation, scavenging free radicals or by increasing superoxide dismutase (SOD) and catalase (CAT) activities [111]. The cell viability, wound healing activity and genotoxicity of *S. platensis* were examined, and the results were reported with 0.1% and 0.05% concentration showed a significant effect on L929 fibroblast cell line proliferation. Fibroblast are responsible for inflammation and scar formation during wound healing. Additionally, an incorporated skin cream with 1.125% *S. platensis* extract showed the highest proliferative effect on skin cells [179].

Mycotoxins, including trichothecenes and fumonisins, can also be involved in increasing oxidative stress. In addition, the main active compound phycocyanin is immunomodulatory and anti-inflammatory. It stimulates the production of antibodies and up- or downregulates the genes encoding cytokines [111]. As a result, all these drawbacks of using synthetic cosmetics have resulted in herbal cosmetics that have many benefits to preserving the health of the skin and enhancing its appearance [180]. Phenolic and flavonoid extracts from *Oscillatoria* sp., *Chroococcidiopsis thermalis*, *Leptolyngbya* sp., *Calothrix* sp. and *Nostoc* sp. have antioxidant activity [167]. Lycopene, which was found in *Anabaena vaginicola* and *Nostoc calcicola*, also showed antioxidant effects [181]. Polysaccharides from *Nostac flagelliforme* are used as free-radical scavengers [182]. A hot water extract of *Nostochopsis* sp. caused the inhibition of the tyrosinase enzyme [183] and displayed a major role in melanin synthesis [184] as it reduced α-melanocyte-stimulating hormone-induced melanin synthesis in B16 mouse melanoma cells and by acid and alkaline treatment [168]. Sacran, a novel sulfated polysaccharide, was extracted from *Aphanothece sacrum* and its anti-inflammatory activity was assessed [185]. Morone et al. [39] reported the bioactive potential of cyanobacteria that summarized the effects of aqueous and organic extracts from different species, MAAs, carotenoids, EPS, SKY and C-phycocyanin on anti-inflammatory, antioxidant, antiaging, moisture absorption and retention photoprotection and the whitening of the skin for cosmetics and cosmeceuticals, which were examined using different assays. Furthermore, the contents of the carotenoids and chlorophyll in the ethanolic extracts from the cyanobacteria species were determined by HPLC-PDA and employing the colorimetric tool of Folin–Ciocalteu to measure the total phenolic contents showed a dry biomass in mg GAE g^−1^, where the highest phenolic content of *S. salina* LEGE 06099 was reported as (2.45 mg GAE g^−1^) (*p* < 0.05), then *Phormidium* sp. LEGE 05292 exhibited (1.52 mg GAE g^−1^) and *Cyanobium sp*. LEGE 06113 displayed (1.41 mg GAE g^−1^) [38]. The carotenoid and chlorophyll utilized as antioxidant and free radical scavenging agents, could be used as well as skin antiaging and skin protection candidate against UV-induced photo-oxidation. Ultimately, with the increase in demand for natural products for body, skin, health and welfare treatments in spa and thalassotherapy centers, cyanobacteria may be seen as natural and ecofriendly sources from a significant bioactive constituent with advantageous effects for skin health, for the development of cosmetics industry investment. Therefore, there is a call for the promotion of research into cyanobacteria ingredients and their implications.

## 4. Total Synthesis and Stereochemical Determination of Marine Cyanobacteria Bioactive Compounds

**Figure 11:**
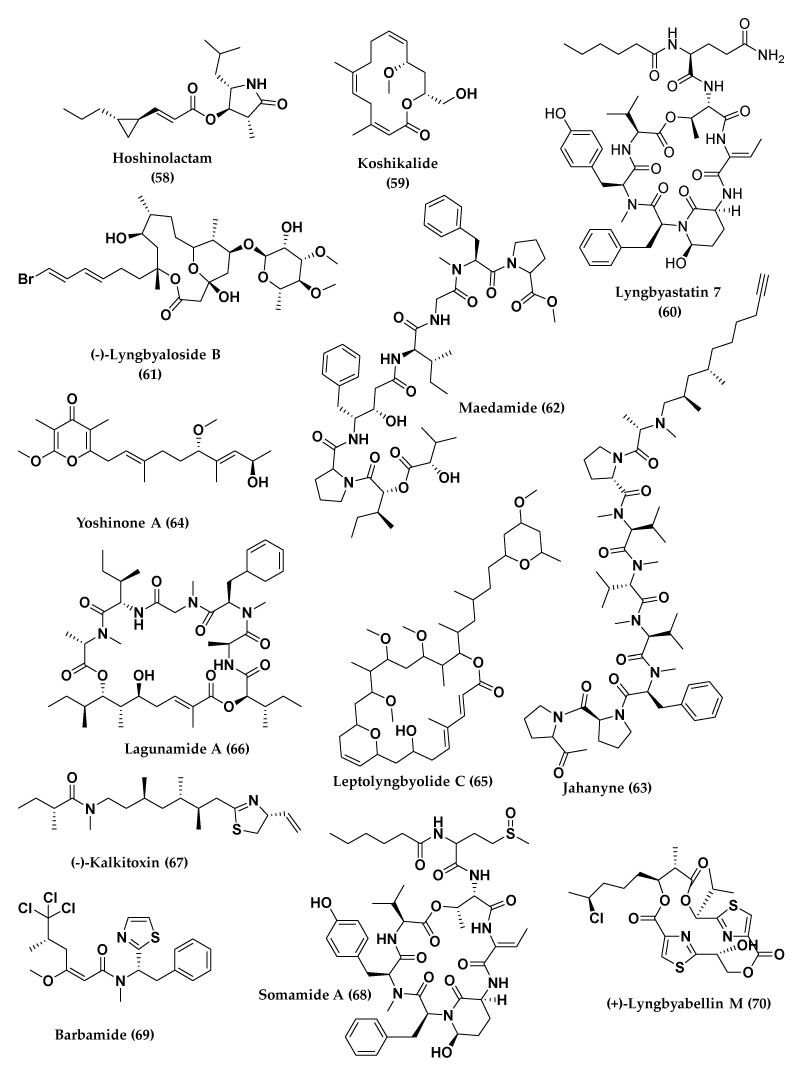
Structures of synthesized compounds (**58**–**70**) isolated from marine cyanobacteria.

**Figure 12:**
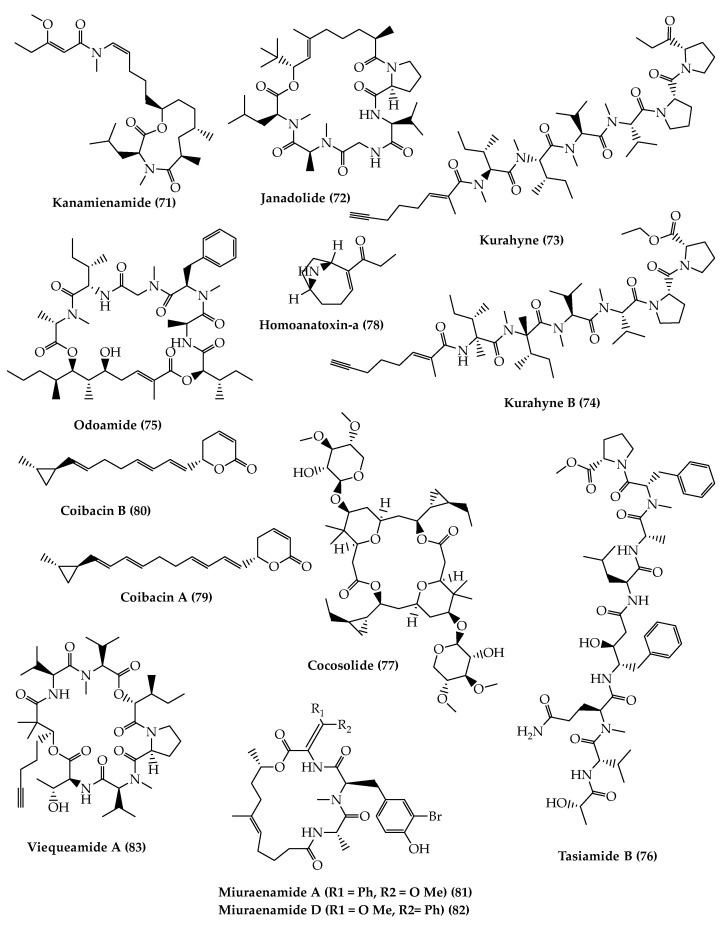
Structures of synthesized compounds (**71**–**83**) isolated from marine cyanobacteria.

Owing to their remarkable variety of structures and fascinating biological behavior, marine cyanobacteria have received exceptional interest from the scientific community [186]. While all of these are marine cyanobacteria advantages, the difficulty in the cultivation and processing of cyanobacteria and their resistance to laboratorial cultivation make it difficult to extract large quantities of natural products due adolescent constituents in species (i.e., 1 mg of 600 g cyanobacterium) [187]. Likewise, biological activities have not yet been investigated for the same purposes, including animal studies [188]. These issues can be addressed by successful large-scale processing by means of the synthesis of natural marine products, demonstrating a variety of ways to examine their biological activities [187]. Consequently, the overall synthesis of natural marine products has received much interest. Depsipeptides and polyketides are the most popular classes known for their synthesis and structure determination. Here, we are just highlighting one example from each group and their total synthetic route, as shown in Table 2. The most synthesized compounds and their structures were shown in Figure 11 and Figure 12.

### 4.1. Depsipeptides

**Scheme 1:**
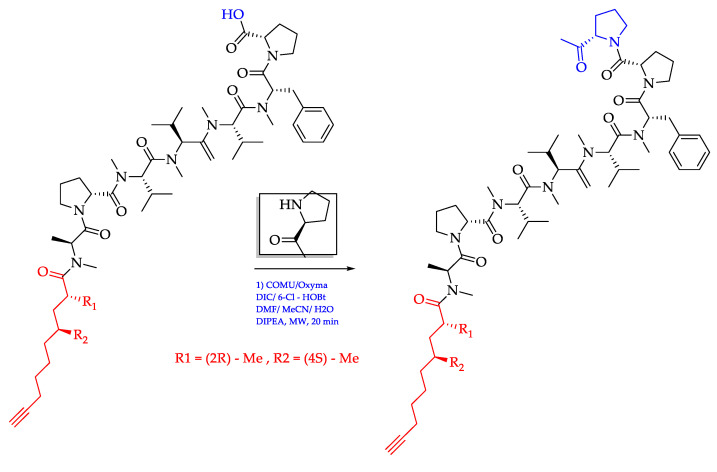

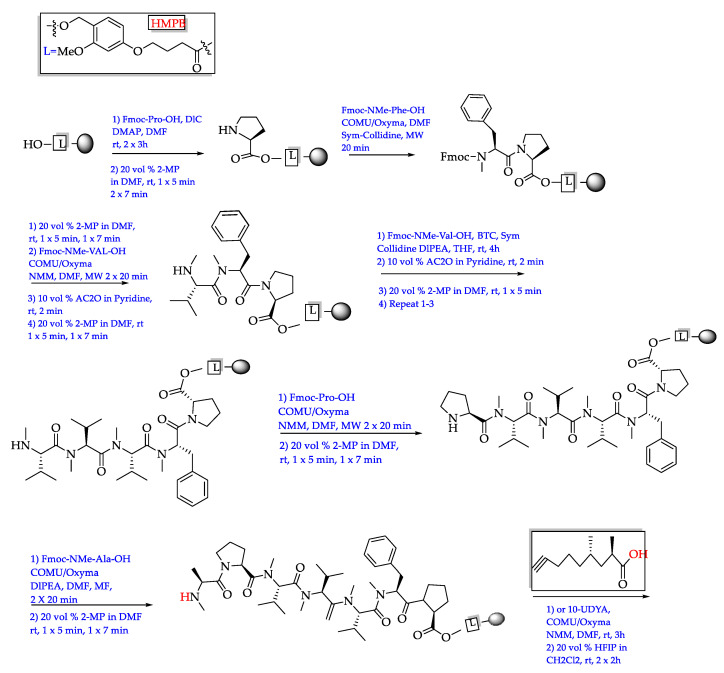
Total Synthesis of jahanyne.

Depsipeptides are natural polypeptides in which one or more of their amides is substituted with a hydroxy acid ester bond that is formed in the core ring structure. They come mainly from marine organisms, especially cyanobacteria [221]. It is interesting to note that various natural cyclic depsipeptides have both special structures and intriguing biological properties, such as antitumor, antifungal, antiviral, antibacterial, anthelmintic and antimicrobial properties. In particular, the strong effects of cyclic depsipeptides on tumor cells have resulted in a variety of clinical trials testing their chemotherapy potential [222]. Depsipeptides have been isolated from some of the most common marine animals, including *Lyngbya majuscula*, *L. confervoides*, *L. bouillonii* and *Rivularia* sp., as shown in Table 2. The cyclic lipodepsipeptide called **41** was isolated from the marine cyanobacterium *L. majuscula* was the subject of a complete synthesis of its isolated form by Gerwick and his coworkers. The EC_50_ = 20.1 ± 6.4 nM showed high ichthyotoxicity and neurotoxicity [187,205]. The full synthesis of jahanyne (**63**), a high-N-methylated lipopeptide containing acetylene, isolated from the marine cyanobacterium *Lyngbya* sp., induced a significant growth inhibition of both HL60 cells and HeLa, with IC_50_ values of 0.63 μM and 1.8 μM, respectively [198]. Scheme 1 displays the complete jahanyne synthesis. In general, the highly N-methylated acetylene containing lipopeptides has a wide range of antitumor, antibiotics and antifungal activities; thus, the chemical synthesis of this subfamily of lipopeptides is very important and can lead to new pharmaceutical discoveries [223]. The total synthesis of koshikalide (**59**) has also been completed, a 14-piece macrolide containing three olefines. The entire stereochemistry was developed to compare the different optical rotations of natural and synthesized koshikalides [224]. In 2010, compound **59** was isolated from a marine cyanobacterium *Lyngbya* sp., assembled in Koshika Prefecture, Shima City, Mie based on spectroscopic analyses, and its relative stereochemistry was created. It showed weak cytotoxicity with an IC_50_ value of 42 µg/mL against HeLa cells [190]. However, its complete stereochemistry could not be elucidated due to the scarcity of the sample (0.3 mg). So, the first total synthesis of koshikalides was performed to clarify the complete stereochemistry of koshikalides, as seen in Scheme 1.

### 4.2. Polyketides Peptide

**Scheme 2:**
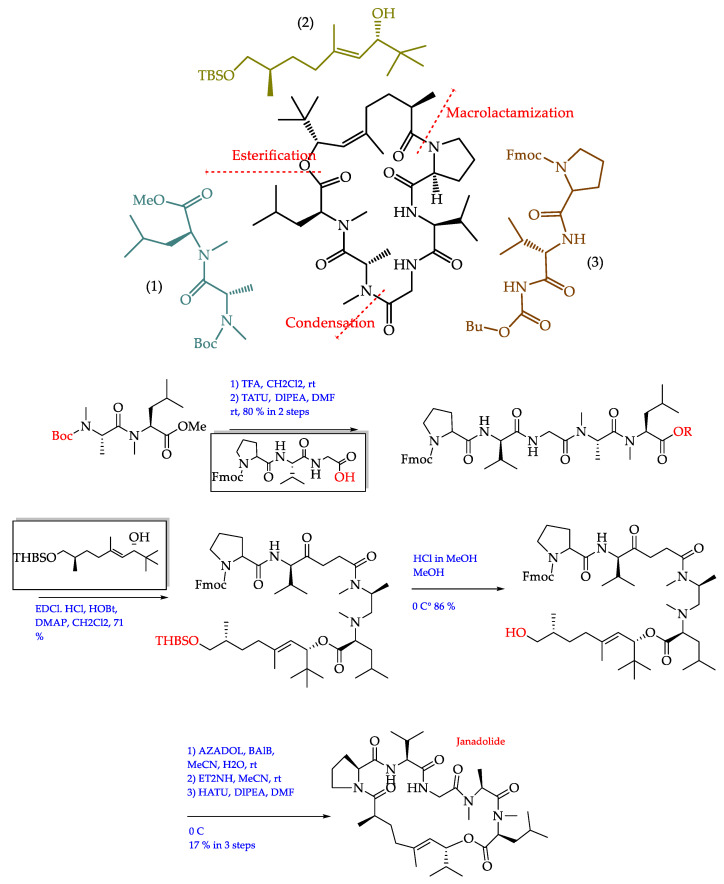
Retrosynthetic analysis and total synthesis of janadolide.

The polyketide natural products are class of compounds that display a magnificent range of functional and structural diversity including antibiotic, anticancer, antifungal, antiparasitic and immunosuppressive properties [225]. So, scientists became concerned with these molecules and have done their best to assemble them [226]. Polyketides are separated from some of the most common species of cyanobacteria, such as *Okeania* sp., *Symploca* sp., *Oscillatoria* sp. and *Paraliomixa miuraensis*, which possess different biological activities, as shown in Table 2. For example, the total synthesis of janadolide (**72**), isolated from an *Okeania* sp. Compound **72** showed potent antitrypanosomal activity with an IC_50_ value of 47 nM, without cytotoxicity against human cells [210]. The steps of the total synthesis of **72** were due to the macrolactamization of the proline moiety and fatty acid moiety manifested by the amide bond, as seen in Scheme 2 [227]. Kurahyne B (**74**), a new kurahyne analog, has been separated from the marine cyanobacterium *Okeania* sp. collected in March 2013 at a depth of 0–1 m close to Jahana, Okinawa Prefecture, Japan. Its gross structure was elucidated using UV, IR, 1D and 2D NMR and HRESIMS spectroscopic analyses. The survival and proliferation of the cell lines (namely, the Hela and HL60 cells) was suppressed by compound **74**, with IC_50_ values of 8.1 and 9.0 μM, respectively, whereas kurahyne B and kurahyne generate the same growth inhibition effect. The primary total synthesis of **73** was also accomplished [186]. Compound **73** is a novel acetylene-containing lipopeptide that was isolated from a marine cyanophyta *Lyngbya* sp. gathered in 2014. It has the same effect as **74**, with IC_50_ values of 8.1 and 9.0 μM, respectively [228]. The absolute configuration was established by the total synthesis of **74** (3.6% overall yield in 14 steps). Additionally, the first total synthesis of **74** was also achieved (3.3% overall yield in 14 steps) [186].

## 5. Conclusions

Herein we are studying 91 compounds; 63 naturally occurring metabolites and 28 compounds synthesized from marine cyanobacteria. According to the best of our knowledge, the 28 synthesized compounds in this article have demonstrated important activities, including antibiotic, anticancer, antifungal, antiviral, anthelmintic, antimicrobial, antiparasitic, and immunosuppressive activities. These compounds have been isolated from the *Lyngbya*, *Oscillatoria*, *Moorea*, *Okeania*, *Symploca*, *Rivularia*, and *Paraliomixa* genera, and include molecules classified as depsipeptides and polyketides. However, the *Lyngbya* genus is associated with a polyphylla group which has had its taxonomic role revised. The potential for the discovery of new natural molecules and biosynthetic pathways associated with new cyanobacteria remains important and requires systematic exploration.

The naturally occurring metabolites were found in various species of 14 genera; *Arthrospira*, *Lyngbya*, *Nostoc*, *Scytonema*, *Rivularia*, *Calothrix*, *Dapis*, *Okeania*, *Moorea*, *Cyanobium*, *Leptolyngbya*, *Symploca*, *Anabaena*, *Aphanothece*, *Oscillatoria*, *Paraliomixa*. These metabolites can be categorized into eight chemical groups (including lipopeptides, polyketides, peptolide, depsipeptides, peptides, protein, polysaccharide and alkaloids) most of which are peptide by-products (over 70% of the families). No strong relationships were observed globally the between chemical groups and the specificity of the various types of bioactivity.

Clinically, we found prospective biomedical or behavioral research studies on 8 compounds/drugs and 4 as synthetic analogs—**47**, **48** and **59** derived from **45**, and **46** derived from **44** isolated from marine cyanobacteria, which are designed to treat different diseases including treatments of different kinds from cancer, among them sarcoma, leukemia, lymphoma, liver, lung, kidney, prostate, and ovarian cancer.

Further in vivo studies remain necessary to precisely comprehend the mechanisms of action associated with cyanobacterial metabolites. For example, nostoflan exhibited potent antiviral activity against herpes simplex virus type 1 (HSV-1). Compound **40** displayed a similar effect on the human immunodeficiency virus type 1 with an IC_90_ of 1 mM employing cellular and cervical explant models. The inhibition of in vitro human viruses’ replication, including HCMV, HSV-1, HHV-6 and HIV-1, was impacted by the supplement of the broad-spectrum antiviral calcium spirulan. Taken together, these indicators resonate the potential notion regarding the role of the marine products in fighting of coronavirus [229], and thus warrant insight investigations to test the marine secondary against SARS-CoV-2 and particularly to face the COVID-19 pandemic.

## Figures and Tables

**Table 1:** Cyanobacterial derived natural products used in clinical tests.

Compound Name (No.)/Chemical Class	Cyanobacteria Species/Source	Type of Activity	Clinical Status/Study Type	References
Dolastatin 10 (**44**)/Depsipeptides	*Symploca* sp.	Sarcoma, Leukemia, Lymphoma, Liver Cancer, and Kidney Cancer, among others.	Drug Investigational	[92,93,94,95,96,97,98,99,100]
		Leukemia Lymphoma	Drug Intervention Phase II	
		Prostate Cancer	Drug Intervention Phase II	
		Kidney Cancer	Drug Intervention Phase II	
		Extrahepatic Bile Duct Cancer Gallbladder Cancer Liver Cancer	Drug Intervention Phase II	
		Ovarian Cancer Sarcoma	Drug Intervention Phase I	
		Leukemia Myelodysplastic Syndromes	Drug Intervention Phase I	
		Pancreatic Cancer	Drug Phase II Intervention	
Cemadotin (**47**), Tasidotin (**48**) and Synthadotin (**49**) (Derived from dolastatin 15 (**45**)/Depsipeptide	*Dolabella auricularia* and cyanobacteria *Symploca* (later)	Melanoma	Drug: ILX651 Intervention Phase II	
		Hormone-refractory Prostate Cancer		[101,102,103,104]
		Non-Small-Cell Lung Carcinoma		
Cryptophycin 52 (**43**)/(Synthetic analog of cryptophycin 1 (**5**) Depsipeptides	*Nostoc* sp., *terrestrial* cyanobacteria	Schizophrenia	Cognitive remediation therapy, Intervention Not Applicable Phase	[101,105,106]
		Hypertension Metabolic Disorder	Drug: losartan potassium (+) hydrochlorothiazide, Intervention Phase III	
Toyocamycin (**52**)/Pyrrolopyrimidine nucleoside	*Streptomyces toyocaensis* Cyanobacteria	Non-Small-Cell Lung Carcinoma	Drug Experimental	[101,107]
Phytoalexin (**53**)/Polysaccharides	*Scytonema ocellatum*	Type 2 Diabetes (RED)	Drug Phase *I* Intervention	[108,109]
Soblidotin (**46**)/(Synthetic analog of dolastatin 10) Depsipeptides		Sarcoma	Drug Intervention Phase II	[101,110]
		Lung Cancer		
Phycocyanin (**54**)/A pigment-protein complex	Spirulina	Chronic Periodontitis	Drug: Spirulina capsules, Intervention Phase IV	[111,112,113]
		Metabolic Syndrome	Dietary Supplement: Spirulysat^®^ Dietary Supplement: Placebo Intervention Not Applicable Phase	
Anatoxins-a (**51**)/ Peptides	*Anabeana circinalis*	Amyotrophic Lateral Sclerosis	Recruiting Patient Registry Intervention	[114,115]
Bacteriocins/Peptides	43 different cyanobacteria viz., *Prochlorcoccus marinus*, *Synechococcus* sp., *Cyanothece* sp., *Microcystis aeruginosa*, *Synechocystis*, *Arthospira*, *Nostoc*, *Anabaena*, *Nodularia*	Ventilator Associated Pneumonia	Lactobacillus bacteria Intervention	[116,117,118,119,120]
		Colic, Infantile Probiotic Gut Microbiome Bifidobacterium Breve	Drug Intervention Phase IV	
		Healthy	Plantaricin A—rejuvenating cream, antioxidant serum, rejuvenating serum Intervention Phase III	
		White Spot Lesion of Tooth Long Term Adverse Effects Caries, Dental Orthodontic Appliance Complication	Drug: Probiotic Toothpaste Drug: Dr. Reddy’s Clohex Other: Control Group Intervention Phase I and II	
Curacin (**50**)/Lipopeptides	*Lyngbya majuscule*		In vivo animal trails. Preclinical Phase (but it served as a lead compound)	[91]
Cyanovirin-N (**40**) (CVN)/A protein	*Nostoc ellipsosporum*	Inhibiting HIV cell entry in a highly specific manner.	Preclinical Phase	[121]

**Table 2:** List of synthesized compounds isolated from marine cyanobacteria sources and their activities.

Marine Source	Compound Name/Class	Region/Year	Biological Activity	References
Marine cyanobacterium	Hoshinolactam (**58**)	The coast near Hoshino, Okinawa/2017	Antitrypanosomal activity, IC_50_ = 3.9 (Syn.), 6.1 (Nat.) nM. Cytotoxicity against MRC-5 cells IC_50_ > 25 μM (Syn. and Nat.) PC = pentamidinea NC = not (in vitro)	[189]
*Lyngbya* sp.	Koshikalide (**59**)	Koshika, Shima City, Mie prefecture/2010	Cytotoxicity against HeLa S3 cells, IC_50_ = 42 µg/mL. PC = not NC = not (in vitro)	[190]
*Lyngbya majuscule*.	Apratoxin A (**22**)/Cyclodepsipeptide	Finger’s Reef, Apra Harbor, Guam/2001	Cytotoxicity against KB (IC_50_ = 0.52 nM) and LoVo cancer cells (IC_50_ = 0.36 nM). (in vitro) Against a colon tumor and ineffective against a mammary tumor. (in vivo)	[191,192]
*Lyngbya* sp. & *Lyngbya confervoides*./	Lyngbyastatin 7 (**60**)/Lariat-type cyclic depsipeptide	Mangrove channel, Kemp Channel, at the northern end of Summerland Key, Florida Keys/2005	Blocking elastase activity, IC_50_ = 70 nM, antiproliferation and abrogating the elastase-triggered induction of proinflammatory cytokine expression. PC = sivelestat, or (DMSO) NC = NR (in vivo)	[193,194]
*Lyngbya bouillonii*	(−)-Lyngbyaloside B (**61**)/Glycoside macrolide	Ulong Channel, Palau/2000	Cytotoxicity against KB cells, IC_50_ = 4.3 µM and LoVo cells, IC_50_ = 15 µM.	[195,196]
*Lyngbya* sp.	Maedamide (**62**)/Acyclic peptide	Kuraha, Okinawa/2014	Inhibitory activity against chymotrypsin, IC_50_ = 45 μM, HeLa and HL60 cells, IC_50_ = 4.2 and 2.2 μM.	[197]
*Lyngbya* sp.	Jahanyne (**63**)/Lipopeptides	The coast near Jahana, Okinawa, Japan/2015	Cytotoxicity against HeLa cells and HL60 cells, IC_50_ = 1.8 μM and 0.63 μM (et al., 2015) natural jahanyne, IC_50_ = (22 ± 2, 4.6 ± 1.2 μM) and synthetic (21 ± 2, 8.3 ± 2.3 μM).	[198,199]
*Leptolyngbya* sp.	Yoshinone A (**64**)	Ishigaki island, Okinawa, Japan/2014	Antiobesity activity (in vivo) in mice, (Inhibited differentiation of 3T3-L1 cells into adipocytes, EC_50_ = 420 nM) and toxicity against *Saccharomyces cerevisiae* ABC16-Monster, (IC_50_ = 63.8 µM).	[200]
	Leptolyngbyolide C (**65**)/Macrolide	On the coast of Itoman City, Okinawa, Japan/2007	Growth-inhibitory activity against HeLa S3 cells, (IC_50_ = 0.64 µg mL^−1^) and depolymerization of F-actin (EC_50_ = 26.9 µg mL^−1^). (in vitro)	[201]
*Lyngbya majuscula*	Lagunamide A (**66**)/Cyclodepsipeptide	Western lagoon of Pulau Hantu Besar, Singapore/June 2007	Antimalarial activity against *Plasmodium falciparum*, IC_50_ = 0.19 and cytotoxic activity against P388 murine leukemia cell lines, IC_50_ = 6.4 nM, and moderate antiswarming activities against *Pseudomonas aeruginosa* PA01. PC: MeOH-treated plate	[202,203]
*Lyngbya majuscula*	(−)-kalkitoxin (**67**)	Curaçao/2004	Cytotoxicity against the human colon cell line HCT-116, IC_50_ = 1.0 × 10^−3^ μg mL^−1^, inhibited hypoxia-induced activation of HIF-1 in T47D breast tumor cells (IC_50_ = 5.6 nM)	[204]
*Lyngbya majuscula*	Antillatoxin (**41**)/Cyclic lipodepsipeptide	Curacüao/2005	Strong ichthyotoxicity and neurotoxicity (EC_50_ = 20.1 ± 6.4 nM).	[187,205]
*Lyngbya majuscula* & *Schizothrix* sp.	Somamide A (**68**)/Macrocyclic depsipeptide	Fijian Island/2005		[187]
*Lyngbya majuscula*	Barbamide (**69**)/Lipopeptide	Curacüao/1996	Potent molluscicidal activity against *Biomphalaria glabrata*, LC_100_ = 100 µg/mL	[206,207]
*Moorea bouillonii*	(+)-Lyngbyabellin M (**70**)/Lipopeptide	North lagoon at Strawn Island, Palmyra Atoll, USA/August 2009	Not reported	[208,209]
	Kanamienamide (**71**)	The shore of Kanami, Kagoshima, Japan/2016	Growth-inhibitory activity. As a necrosislike cell death inducer.	[210]
*Okeania* sp.	Janadolide (**72**)/Cyclic polyketide- peptide	Bise, Okinawa Prefecture, Japan/2016	Antitrypanosomal activity without cytotoxicity against human cells (IC_50_ 47 nM)	[211]
	Kurahyne (**73**) (N-Me) Kurahyne B (**74**) (N-H)	The coast near Jahana, Okinawa/March 2013	Growth-inhibitory activity (Inhibited the growth of both HeLa and HL60 cells, IC_50_ = 8.1 and 9.0 μM) PC = Adriamycin	[186]
	Odoamide (**75**)/Cyclodepsipeptide	Odo, Okinawa Prefecture, Japan/May 2009	(in vitro) Cytotoxicity against HeLa S3 cells, IC_50_ = 26.3 nM. Toxicity against brine shrimp (Artemia), LD_50_ = 1.2 µM.	[212]
*Symploca* sp.	Tasiamide B (**76**)/Acyclic peptide	Micronesia by Moore et al., 2003	Cytotoxic against KB cells, IC_50_ = 0.8 µM	[213]
	Cocosolide (**77**)/Glycosylated macrolide	Cocos Lagoon and Tanguisson reef flat, Guam/2016	Inhibited IL-2 production in both T-cell receptors also suppressed the proliferation of anti-CD3-stimulated T-cells in a dose-dependent manner. (IC_50_ > 50 mm).	[214]
*Oscillatoria Formosa*	Homoanatoxin-a (**78**)	Inniscarra reservoir, County Cork, Ireland/2004	Cytotoxic activity LD_50_’s in mice of 200–250 µg/kg.	[215]
*Oscillatoria* sp.	Coibacin A (**79**)/Unsaturated polyketide lactone	Panamanian/2012	Antileishmanial activity against axenic amastigotes of *Leishmania donovani* (IC_50_ = 2.4 μM). Cytotoxicity against NCI-H460 cells (IC_50_ = 31.5 μM). Antiinflammatory activity by cell-based nitric oxide (NO) (IC_50_ = 20 μM).	[216,217]
	Coibacin B (**80**)/Unsaturated polyketide lactone		As a leishmanicidal drug (IC_50_ = 7.2 μM); cytotoxicity against human cancer lung cell lines (NCI-H460), IC_50_ = 17.0 μM. Active coibacin representative (IC_50_ = 5 μM).	
*Paraliomixa miuraensis*	Miuraenamide A (**81**) (R1 = Ph, R2 = O Me) Miuraenamide D (**82**) (R1 = O Me, R2= Ph)/Cyclodepsipeptides	The seashore on Miura Peninsula in Kanagawa, Japan by Ojika et al., 2006	Cytotoxicity against HeLa cells, IC_50_ = A (0.031), D (0.021) μM. Against HeLa-S3 cell line, IC_50_ = A (0.38), D (1.32) μM. antiphytophthora activity 3, 30 ng/disk	[218]
*Rivularia* sp. “button” Marine cyanobacterium	Viequeamide A (**83**)/Cyclic depsipeptide	Near the island of Vieques, Puerto Rico/2012	Highly toxic against H460 human lung cancer cell lines, IC_50_ = 60 nm. PC = paclitaxel (3.2 nM) and etoposide (63.1 nM)	[219,220]

PC (positive control) and NC (negative control).

## Data Availability

Not applicable.
